# The development and validation of the awareness and knowledge of diabetes distress questionnaire among doctors in Malaysia

**DOI:** 10.1371/journal.pone.0272658

**Published:** 2022-08-10

**Authors:** Grace Jikinong, Pauline Siew Mei Lai, Ahmad Ihsan Abu Bakar, Tun Firzara Abdul Malik

**Affiliations:** 1 Faculty of Medicine, Department of Primary Care Medicine, University of Malaya, Kuala Lumpur, Malaysia; 2 Hospital Pusrawi Sdn Bhd, Jalan Tun Razak, Kuala Lumpur, Malaysia; Universiti Sains Malaysia Institut Perubatan dan Pengigian Termaju, MALAYSIA

## Abstract

The main objective of this study was to develop and validate the Awareness and Knowledge of Diabetes Distress (AKODD) questionnaire, so that it can be used to assess the knowledge attitude and practice of doctors who treat patients with diabetes distress. This validation study was conducted at the University Malaya Medical Centre, Kuala Lumpur, Malaysia from June to July 2019. Doctors from the Departments of Primary Care Medicine, Medicine, Psychological Medicine, Emergency Medicine and Staff Health Unit, who could understand English were recruited, as they treat patients with diabetes or diabetes distress. The AKODD was developed based on literature review. Next, an expert panel met to review findings from literature and to develop the items for AKODD. The AKODD has 3 sections: socio-demographic information, awareness and knowledge. It was then piloted among 7 doctors from the Departments of Primary Care Medicine, Medicine, Psychological Medicine and Emergency Medicine. No problems were encountered. Hence, no changes were made, and the AKODD was administered twice: at 0 and 2 weeks as part of the validation process. Discriminative validity was assessed by comparing scores of doctors who had/had not attended a diabetes course before. A total of 103/119 doctors agreed to participate (response rate = 86.6%). Flesch Reading Ease was 51.1. Thirty-three doctors (32.0%) have heard of diabetes distress before. Doctors had a good level of knowledge regarding diabetes distress with a median score of 77.8% (IQR:66.7–88.9). The AKODD had adequate discriminative validity between participants who had (83.3%)/had not attended a diabetes course before (72.2%; p<0.049). The AKODD had good internal consistency (Kuder-Richardson = 0.931) and adequate reliability as 9/18 items were not statistically significant at test-retest. The AKODD was found to be a valid and reliable questionnaire to assess the awareness and knowledge of diabetes distress among doctors in Malaysia as it had adequate psychometric properties.

## Introduction

In 2014, the World Health Organization (WHO) estimated that there were 422 million adults diagnosed with diabetes [1]. In Malaysia, 1 in 5 adults aged ≥18 years in Malaysia have type 2 diabetes mellitus (T2DM). This means that 3.9 million individuals in Malaysia have T2DM [2], of which the prevalence is highest amongst the Indians (24.9% in 2011 and 19.9% in 2006), followed by the Malays (16.9% in 2011 and 11.9% in 2006), and the Chinese (13.8% in 2011 and 11.4% in 2006) [3].

Diabetes is a complex chronic disease that is demanding and challenging for patients to manage [4]. Managing diabetes requires constant patient attention, active self-management, as well as support from family, friends and health care providers [4]. All these requirements can cause a patient to be overwhelmed, frustrated, angry or discouraged; leading to a huge negative impact on the patient’s psychological well-being [5]. These emotional burdens and worries of being diagnosed with diabetes, fear of its possible complications, negative emotional reactions towards the demanding management of diabetes, and concerns of unsupportive family, friends and health care providers is known as “diabetes distress” [6].

Diabetes distress can negatively affect a patient’s self-management and glycaemic control, which further causes poorer diabetes outcomes, if it is not addressed [7]. A recent meta-analysis of 55 studies reported an overall prevalence of 36% for diabetes distress in people with T2DM [8]. Prevalence of diabetes distress was found to be significantly higher in females, as well as in people with a higher prevalence of comorbid depressive symptoms [8].

Several questionnaires have been developed and validated to screen for diabetes distress among patients, such as the Diabetes Distress Scale (DDS-17) [5] and the Problem Areas in Diabetes Scale (PAID) [9]. These questionnaires assess the severity of a person’s distress level and helps identify the source of distress. However, diabetes distress remains largely undetected and undiagnosed by doctors as they are unable to identify, assess and provide support for a patient’s psychological problems [10]. A search of published literature revealed that there is currently no validated questionnaire to assess the awareness and knowledge among doctors regarding diabetes distress. Hence, our study aimed to develop and validate a questionnaire to assess the awareness and knowledge regarding diabetes distress among doctors in Malaysia.

## Materials and methods

### Phase 1: Development of the awareness and knowledge of diabetes distress (AKODD) questionnaire

The AKODD was developed in June 2019 based on literature review (which was limited to English) and reviewed by an expert panel (which consisted of four family medicine specialists, two psychiatrists, one endocrinologist, one academic pharmacist and one family medicine master candidate). This process was reiterative until the expert panel was satisfied with the questionnaire. The AKODD was then pilot tested among doctors who treat patients with diabetes.

### Phase 2: Validation of the awareness and knowledge of diabetes distress (AKODD) questionnaire

This validation study was conducted from June to July 2019, at the University Malaya Medical Centre, Kuala Lumpur, Malaysia. Participants were doctors from the departments of Primary Care Medicine, Medicine, Psychological Medicine, Emergency Medicine and Staff Health Unit, as they treat patients with diabetes. Doctors from Psychological Medicine were also included as patients with diabetes who experience diabetes distress or psychological problems like depression would be referred to them. Doctors who were on long leave and not fluent in English were excluded.

Sample size was calculated based on the rule of thumb of items to participant ratio of 1:5 to perform factor analysis [11]. There are 18 items in the AKODD. Hence, the total number of participants required was (18*5) 90. However, an additional 20% doctors were recruited to account for the drop out rate at retest.

Convenience sampling was used to recruit participants. Participants were approached after departmental teaching sessions. The aim of this study was explained to participants using the participant information sheet. Written informed consent was obtained from those who agreed to participate. Participants were then asked to answer the AKODD questionnaire at 0 and 2 weeks later. Ethics approval was obtained from the Medical Ethics Committee, University Malaya Medical Centre (MED ID: 2019527–7446).

#### Patient and public involvement

No patients were involved in the development of the AKODD questionnaire as this questionnaire was only administered to healthcare professionals.

#### Data analysis

Data was analysed using IBM Statistical Package for the Social Sciences (SPSS) software version 25 (Illinois, United States of America). The Kolmogorov-Smirnov test was used to assess for normality. Non-parametric tests were used as data was found to be not normally distributed. Descriptive statistics were used to analyse categorical variables and findings were reported in frequencies and percentages. Medians and interquartile range were used to present continuous variables.

#### Scoring of the awareness and knowledge of diabetes distress (AKODD) questionnaire

The items in the awareness section of the AKODD only provided descriptive data, hence only descriptive analysis was performed without any scoring. The items in the knowledge section of the AKODD had three options: true, false and don’t know. One point was given for a correct answer. Correct answers for each item were based on findings from literature review during the development of the AKODD. No points were awarded for incorrect or don’t know answers. The AKODD was scored by calculating the total amount of correct answers in the knowledge section, which was then converted to percentage in order to calculate the median knowledge score of the participant.

#### Validity

Flesch Reading Ease was calculated to assess the readability of the AKODD [12]. Difficulty factor was calculated by the number of correct answers divided by the total number of participants [13]. Convergent validity was not performed as no validated tool was available during this study period to assess the knowledge and awareness regarding diabetes distress among doctors. For discriminative validity, participants were divided into two groups: participants who have attended a diabetes course before versus those who have never attended a diabetes course. In Malaysia, updates regarding the management of diabetes are organised once a year for early career doctors. Among the topics covered in this one-day course are the psychological aspects of treating a patient with diabetes such as diabetic distress. The Mann-Whitney U test was used to discriminate the knowledge level between participants who have and have not attended a diabetes course before. A p-value of <0.05 was considered as statistically significant.

#### Reliability

Kuder-Richardson Formula 20 was used to assess the internal consistency of the AKODD as responses were dichotomous [14]. Test-retest reliability was determined using the McNemar test [15].

## Results

### Phase 1: Development of the awareness and knowledge of diabetes distress (AKODD) questionnaire

**Fig 1:**
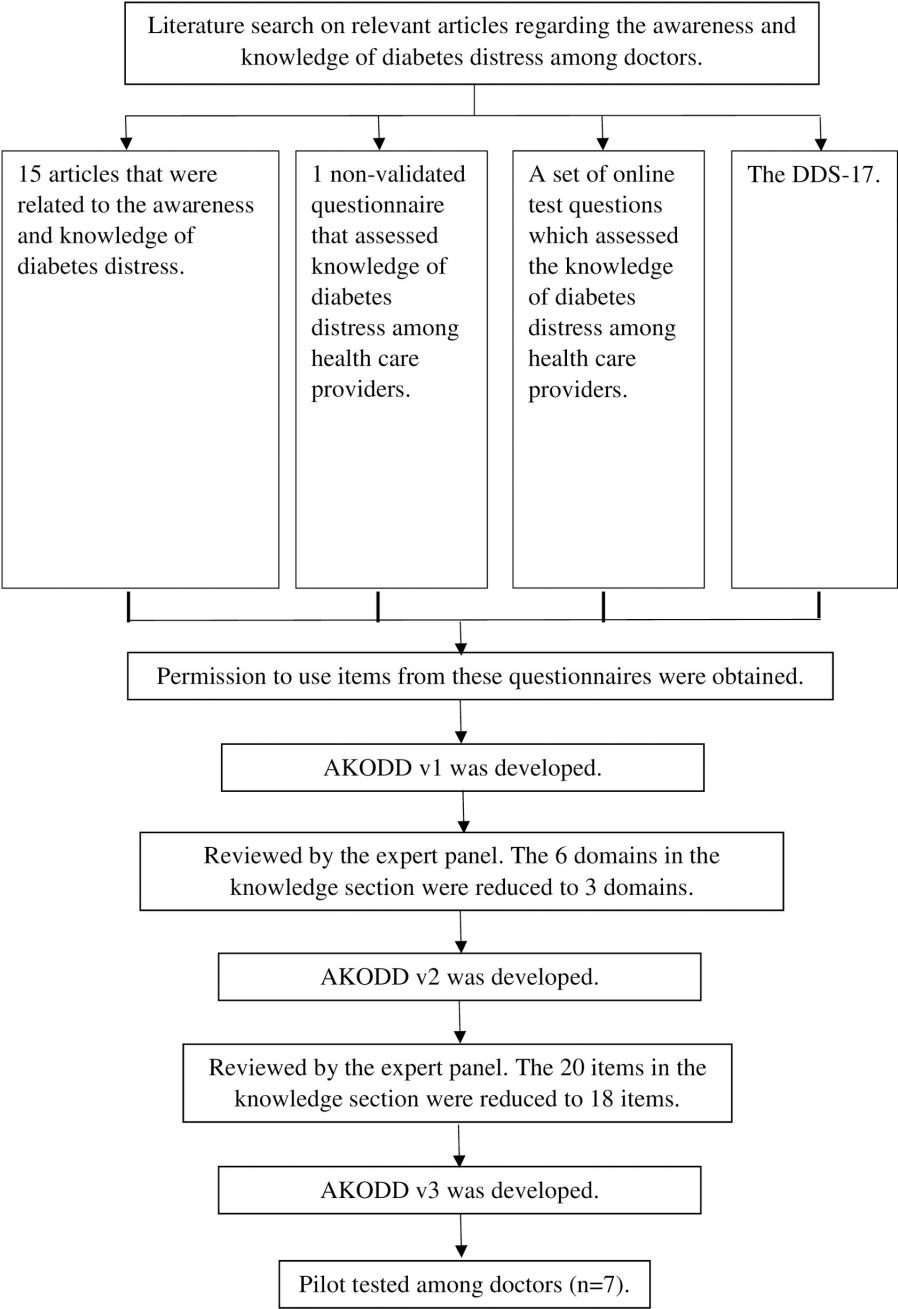
Development of the awareness and knowledge of diabetes distress (AKODD) questionnaire.

The initial literature review led to version 1 of the AKODD. The expert panel met twice to produce versions 2 and 3. The final version of the AKODD (v3) comprised of three sections. Section A had 8 items to collect socio-demographic information, section B consisted of 2 items on awareness of diabetes distress, section C consisted of 18 items on knowledge that were further divided into 3 domains: “diabetes distress in general”, “consequences of untreated diabetes distress” and “diabetes distress management” (Fig 1, S1 File 1).

Only the knowledge section of the AKODD (which consisted of 18 items) could be validated as it contained “true”, “false” or “don’t know” options that could be scored. The remaining sections only provided descriptive data. During the pilot study, participants took about 10 minutes to complete the AKODD questionnaire. No problems were encountered. Hence, no further changes were made.

### Phase 2: Validation of the awareness and knowledge of diabetes distress (AKODD) questionnaire

A total of 103/119 doctors agreed to participate (response rate = 86.6%). The demographic characteristics of participants are shown in Table 1.

**Table 1:** Demographic characteristics of participants (n = 103).

Characteristics	n (%)
**Median age in years** (IQR)	32.0 (30.0–35.0)
**Gender** Male Female	37 (35.9) 66 (64.1)
**Ethnicity** Malay Chinese Indian and others*	39 (37.9) 42 (40.8) 22 (21.4)
**Qualification** Medical officer Specialist	81 (78.6) 22 (21.4)
**Median years of experience as a doctor** (IQR)	5.0 (4.0–9.0)
**Department** Primary care medicine Medicine Psychological medicine Emergency medicine Staff health	28 (27.2) 25 (24.3) 23 (22.3) 22 (21.4) 5 (4.9)
**Median number of diabetes patients seen per day** (IQR)	8.0 (5.0–10.0)
**Have attended a diabetes course**	70 (68.0)

IQR = Interquartile range.

* Others include Punjabi = 2, Myanmar = 1, Ceylonese = 1 and Maldivian = 1.

#### Readability

Flesch reading ease score was 51.1.

#### Difficulty factor

Eleven out of 18 (61.1%) items had a difficulty factor of > 0.7 (“easy” questions), six (33.3%) items had difficulty factors between 0.3–0.7 (“average” questions), and one (5.6%) item had a difficulty factor of < 0.3 (“difficult” question) [Table 2].

**Table 2:** Difficulty factor of each item in the awareness and knowledge of diabetes distress (AKODD) questionnaire.

Domain	No.	Item	Difficulty factor
Diabetes distress in general	C1	Diabetes distress is the emotional burdens and worries that patients experience when they are managing their diabetes.	0.83
	C2	Diabetes distress is another term used to describe depression that patients experience while living with diabetes.	0.34
	C3	Older patients are more likely to develop diabetes distress.	0.14
	C4	Diabetes distress occurs when patients with diabetes feel that they are unable to keep up with the routines of managing their diabetes.	0.83
	C5	Diabetes distress occurs when a doctor does not take a patient’s concerns seriously and does not provide clear enough directions on how to manage a patient’s diabetes.	0.66
	C6	Diabetes distress occurs when a patient with diabetes feels that family or friends do not understand how difficult it is for them to deal with diabetes and are not supportive.	0.76
	C7	Patients experience diabetes distress when they do not understand why their blood sugar levels keep increasing despite eating correctly or adhering to their diabetic medications, and subsequently feel like giving up.	0.73
		**Median domain score (IQR) [range]**	71.4 (57.1–85.7) [0–100]
**Consequences of untreated diabetes distress**	C8	Diabetes distress can lead to poorer control of diabetes.	0.88
	C9	Diabetes distress does not affect a person’s health-related quality of life.	0.82
	C10	Diabetes distress does not affect medication adherence.	0.84
	C11	Diabetes distress may lead to depression.	0.82
	C12	Diabetes distress may lead to poorer self-care (eg: diet, exercise).	0.87
		**Median domain score (IQR) [range]**	100.0 (80.0–100.0) [0–100]
**Diabetes distress management**	C13	Diabetes distress cannot be screened using questionnaires.	0.43
	C14	A patient should be screened for diabetes distress if their glycaemic control remains persistently poor.	0.86
	C15	Diabetes distress must be screened when a patient with diabetes has onset of diabetic complications.	0.66
	C16	Addressing and talking about a patient’s specific areas of concerns in managing their diabetes can help reduce diabetes distress.	0.85
	C17	Diabetes distress is highly responsive to interventions that enhance diabetes self-management.	0.66
	C18	All patients with diabetes distress need to be referred to a psychologist.	0.45
		**Median domain score (IQR) [range]**	66.7 (50.0–83.3) [0–100]
		**Total AKODD score**	77.8 (66.7–88.9) [0–100]

IQR = Interquartile range.

#### Knowledge score

The overall median knowledge score of diabetes distress among doctors was 77.8% [Table 2]. Participants scored the highest in the domain for “consequences of untreated diabetes distress”, followed by “diabetes distress in general”, and least for “diabetes distress management”.

#### Discriminative validity

The AKODD was able to discriminate for 13/18 items. However, items C2, C3, C13, C15 and C17 was not statistically different between participants who attended a course on diabetes versus those who did not. In addition, the median total score for each domain in the AKODD was significantly different in all domains except for the domain “diabetes distress in general” [Table 3].

**Table 3:** The discriminative validity of the awareness and knowledge of diabetes distress (AKODD) questionnaire.

Domain	Item	No. of correct responses, n (%)		Chi-square test / Mann-Whitney U test (p-value)	
		Attended a course on diabetes (n = 70)	Have not attended a course on diabetes (n = 33)		
Diabetes distress in general	C1	63 (90.0)	22 (66.7)	Chi-square test	0.004*
	C2	23 (32.9)	12 (36.4)		0.726
	C3	10 (14.3)	4 (12.1)		0.765
	C4	63 (90.0)	22 (66.7)		0.004*
	C5	51 (72.9)	17 (51.5)		0.033*
	C6	58 (82.9)	20 (60.6)		0.014*
	C7	56 (80.0)	19 (57.6)		0.017*
	Median total domain score (IQR)	71.4 (57.1–85.7)	71.4 (0.0–71.4)	Mann-Whitney U test	0.069
Consequences of diabetes distress	C8	67 (95.7)	24 (72.7)	Chi-square test	0.001*
	C9	63 (90.0)	21 (63.6)		0.001*
	C10	63 (90.0)	23 (69.7)		0.010*
	C11	61 (87.1)	23 (69.7)		0.033*
	C12	66 (94.3)	24 (72.7)		0.002*
	Median total domain score (IQR)	100.0 (100.0–100.0)	100.0 (0.0–100.0)	Mann-Whitney U test	0.021*
Diabetes distress management	C13	33 (47.1)	11 (33.3)	Chi-square test	0.186
	C14	66 (94.3)	23 (69.7)		0.001*
	C15	48 (68.6)	20 (60.6)		0.426
	C16	63 (90.0)	24 (72.7)		0.024*
	C17	49 (70.0)	19 (57.6)		0.214
	C18	37 (52.9)	9 (27.3)		0.015*
	Median total domain score (IQR)	66.7 (50.0–83.3)	66.7 (0.0–83.3)	Mann-Whitney U test	0.047*
	Median total score of AKODD	83.3 (66.7–88.9)	72.2 (2.8–83.3)		0.049*

*significantly different at p<0.05.

#### Reliability

At retest, 78/103 participants responded, as 23 participants were uncontactable, and 2 declined participation (response rate = 75.7%). The overall internal consistency of the AKODD was 0.931. All domains in the AKODD had an overall KR score of > 0.7. In addition, all items in the questionnaire had a corrected item-total correlation of > 0.3, except for one item (C3 = 0.144). Nine out of 18 items were not statistically different at test-retest [Table 4].

**Table 4:** Reliability of the awareness and knowledge of diabetes distress (AKODD) questionnaire.

Domain	No.	Item	Internal consistency			Test-retest		
			Corrected Item- Total Correlation	KR if item deleted	Overall KR	No. of correct responses n (%)		McNemar test (p-value)
						Test (n = 103)	Retest (n = 78)	
**Diabetes distress in general**	C1	Diabetes distress is the emotional burdens and worries that patients experience when they are managing their diabetes.	0.827	0.764	0.826	85 (82.5)	74 (94.9)	0.012*
	C2	Diabetes distress is another term used to describe depression that patients experience while living with diabetes.	0.325	0.847		35 (34.0)	29 (37.2)	1.000
	C3	Older patients are more likely to develop diabetes distress.	0.144	0.858		14 (13.6)	16 (20.5)	0.210
	C4	Diabetes distress occurs when patients with diabetes feel that they are unable to keep up with the routines of managing their diabetes.	0.724	0.780		85 (82.5)	71 (91.0)	0.146
	C5	Diabetes distress occurs when a doctor does not take a patient’s concerns seriously and does not provide clear enough directions on how to manage a patient’s diabetes.	0.607	0.797		68 (66.0)	67 (85.9)	0.001*
	C6	Diabetes distress occurs when a patient with diabetes feels that family or friends do not understand how difficult it is for them to deal with diabetes and are not supportive.	0.766	0.769		78 (75.7)	71 (91.0)	0.001*
	C7	Patients experience diabetes distress when they do not understand why their blood sugar levels keep increasing despite eating correctly or adhering to their diabetic medications, and subsequently feel like giving up.	0.685	0.783		75 (72.8)	71 (91.0)	0.001*
**Consequences of untreated diabetes distress**	C8	Diabetes distress can lead to poorer control of diabetes.	0.847	0.893	0.919	91 (88.3)	72 (92.3)	0.289
	C9	Diabetes distress does not affect a person’s health-related quality of life.	0.751	0.911		84 (81.6)	67 (85.9)	0.774
	C10	Diabetes distress does not affect medication adherence.	0.830	0.893		86 (83.5)	69 (88.5)	0.344
	C11	Diabetes distress may lead to depression.	0.727	0.916		84 (81.6)	72 (92.3)	0.039*
	C12	Diabetes distress may lead to poorer self-care (eg: diet, exercise).	0.833	0.894		90 (87.4)	73 (93.6)	0.219
**Diabetes distress management**	C13	Diabetes distress cannot be screened using questionnaires.	0.436	0.747	0.761	44 (42.7)	57 (73.1)	0.000*
	C14	A patient should be screened for diabetes distress if their glycaemic control remains persistently poor.	0.686	0.693		89 (86.4)	74 (94.9)	0.031*
	C15	Diabetes distress must be screened when a patient with diabetes has onset of diabetic complications.	0.476	0.734		68 (66.0)	58 (74.4)	0.210
	C16	Addressing and talking about a patient’s specific areas of concerns in managing their diabetes can help reduce diabetes distress.	0.699	0.686		87 (84.5)	74 (94.9)	0.039*
	C17	Diabetes distress is highly responsive to interventions that enhance diabetes self-management.	0.522	0.721		68 (66.0)	67 (85.9)	0.003*
	C18	All patients with diabetes distress need to be referred to a psychologist.	0.335	0.775		46 (44.7)	39 (50.0)	1.000
		**Overall KR for the AKODD**			0.931			

KR = Kuder-Richardson

* = p-value < 0.05.

## Discussion

The AKODD questionnaire was developed from literature review and by an expert panel. It was found to be valid and reliable in assessing the knowledge of doctors regarding diabetes distress in Malaysia.

The AKODD had a Flesch Reading Ease score of 51.1. This means that the AKODD can be understood by grade 10 students in the United States [12]. All the participants in our study were doctors who had tertiary education. Hence, they encountered no problems in answering the AKODD.

Eleven out of 18 items had a difficulty factor of > 0.7, indicating that the AKODD was moderately easy to answer. Item C3: “Older patients are more likely to develop diabetes distress” had a difficulty factor of 0.14. Most of our participants answered this item incorrectly as they may have assumed that older people were more prone to having diabetes distress. Instead, a study among 148 African American and White adults with T2DM in the United States found that younger age, less satisfaction with social support, and lower physician trust were associated with higher levels of diabetes distress [16]. This is also seen in another study in the United States that identified high diabetes distress was associated with younger age, being female and higher body mass index [17]. A Malaysian study done among 700 patients with diabetes also concur that high diabetes distress was associated with younger age [18].

Only 32.0% of the doctors in our study were aware of the term “diabetes distress”, which was low. However, we were unable to compare our findings with other studies as this topic has not been studied previously. A possible reason for this low level of awareness could be due to the use of the term “diabetes distress”, which may be a novel term that is only gaining prominence in research over the last decade [19]. Doctors may be aware that their patient with diabetes may be experiencing psychological issues or distress while managing their challenging diabetes; but may not be aware that this condition is called “diabetes distress”. Another possible reason for the low awareness could be that doctors may misdiagnose their patients whom are experiencing diabetes distress with depression instead, as physical symptoms of diabetes distress are similar with symptoms of depression [6].

The total overall median knowledge score of the AKODD was 77.8%, indicating that the majority of participants had fairly good knowledge of diabetes distress. Our findings were similar to a previous study which reported that the knowledge of healthcare providers was 78.9% [20]. In particular, participants were able to score a median score of 100% for the domain of “consequences of untreated diabetes distress”, indicating good knowledge regarding the “consequences of untreated diabetes distress”. This could be because many doctors might know the consequences of distress in any disease, therefore they could easily postulate the consequences of diabetes distress. This was followed by the domain for ‘diabetes distress in general’ (median score = 71.4%), as certain doctors might not be aware of what diabetes distress is and its existence, as evidenced by the findings in the awareness section of the AKODD. However, participants scored the least for the domain “diabetes distress management” (median score = 66.7%) as studies show that not many healthcare providers (23.7%) had actually asked their patients regarding how diabetes has affected their lives [21]. Doctors may also not be knowledgeable or experienced enough about diabetes or its management, as a study found that doctors were unable to identify and asses the psychological problems of patients, therefore unable to provide the psychological support that their patients desired [10]. Otherwise, patients and physicians often have different expectations while managing diabetes [22]. Doctors might be unsure on how to manage a patient with diabetes distress as doctors could be misdiagnosing patients with diabetes of having depression instead of diabetes distress, in view symptoms associated with diabetes distress might be mistaken for symptoms of depression [6].

Thirteen out of 18 items in the AKODD questionnaire were significantly different between participants who had attended a course on diabetes course compared to those who have not. As hypothesized, participants who have attended a diabetes course before does have better knowledge than those who have not attended one.

The AKODD showed good internal consistency with an overall Kuder-Richardson score of 0.931. Only 1 item on the questionnaire had a corrected item-total correlation of <0.3. This might be because the other 6 questions in the same domain (diabetes distress in general) were questions about the definitions regarding diabetes distress and its 4 main sources of distress, whereas item C3 was questioning about the risk factor for diabetes distress instead. However, the removal of this C3 item would only increase the Kuder-Richardson score from 0.826 to 0.858, which was not significant. Hence, this item was retained in the AKODD questionnaire.

Nine out of 18 items were statistically not significant at test-retest, indicating that the AKODD questionnaire has achieved stability. The remaining nine items were significantly different at test-retest. Participants may have answered the items correctly during baseline and may have answered it wrongly during the retest, or vice versa. In this case, participants might have improved knowledge regarding diabetes distress while answering the retest which makes it possible for them to answer correctly on the second occasion. They might have read up about diabetes distress during the baseline and retest baseline interval. This is likely because the percentage of correct responses had increase in all items while comparing baseline and retest scores of the AKODD.

The strength of our study was that we recruited a sufficient number of participants for this validation study and had a good response rate of 86.6%. One of the limitations of this study was that convenience sampling was used to recruit participants. It would have been better to recruit participants randomly as recruitment via convenience sampling may have introduced a bias in the selection of participants. This was because participants that attended departmental teaching sessions might have better knowledge as opposed to those who did not attend teaching sessions.

## Conclusion

The AKODD questionnaire was found to be a valid and reliable questionnaire to assess the awareness and knowledge regarding diabetes distress among doctors as it had adequate psychometric properties. It can now be used to assess the awareness and knowledge of doctors regarding diabetes distress, therefore enabling identification of knowledge gaps which can provide further information in the improvement of diabetes care. Hence, it can be used in clinical practice to improve the quality of diabetes care among patients with diabetes.

## Supporting information

S1 File The awareness and knowledge of diabetes distress (AKODD) questionnaire. (PDF)
