# Supplementary material for: The development and validation of the awareness and knowledge of diabetes distress questionnaire among doctors in Malaysia
Source: PLoS One. 2022 Aug 10;17(8):e0272658. doi: 10.1371/journal.pone.0272658 (PMC9365182; doi:10.1371/journal.pone.0272658)
Supplement: S1 File — (PDF) [file pone.0272658.s001.pdf]

ID Number:			

The Awareness and Knowledge of Diabetes Distress (AKODD) Questionnaire

Please fill in the blank spaces or tick (√) the appropriate box.

SECTION A: SOCIO-DEMOGRAPHIC INFORMATION

A1. Age: ________________ years old

A2. Gender: ☐ Male ☐ Female

A3. Ethnicity: ☐ Malay ☐ Chinese ☐ Indian ☐ Others (please specify):______

A4. Qualification achieved:

☐ Bachelors in Medicine and Surgery (eg: MBBS)

☐ Masters

☐ Others (please specify):______

A5. Number of years of experience as a doctor (post-full registration): _____________ years

A6. Current department / unit:

☐ Primary Care Medicine
☐ Medicine
☐ Psychiatry
☐ Staff Health
☐ Emergency

A7. On average, how many patients with diabetes mellitus do you see in a day?

____________ patients/day

A8. Have you attended a course / workshop about diabetes mellitus before?

☐ Yes ☐ No

ID Number:			

SECTION B: AWARENESS REGARDING DIABETES DISTRESS

B1. Have you heard of diabetes distress before?

□ Yes

□ No **(Please skip B2 and proceed to Section C)**

B2. How did you hear about diabetes distress? **(You may tick more than one answer)**

□ I have managed patients with diabetes distress before

□ A patient who knew about diabetes distress told me

□ A family member told me

□ A friend told me

□ Someone I know has diabetes distress

□ From medical school lectures

□ From postgraduate lectures

□ By attending courses / workshops about diabetes

□ From clinical practice recommendations / guidelines

□ Online website

□ Others (please specify): ____

ID Number:			

SECTION C: KNOWLEDGE REGARDING DIABETES DISTRESS

Please tick (√) one response for each statement:

No.	Questions	True	False	Don't Know
**DIABETES DISTRESS IN GENERAL**				
**C1**	Diabetes distress is the emotional burdens and worries that patients experience when they are managing their diabetes.			
**C2**	Diabetes distress is another term used to describe depression that patients experience while living with diabetes.			
**C3**	Older patients are more likely to develop diabetes distress.			
**C4**	Diabetes distress occurs when patients with diabetes feel that they are unable to keep up with the routines of managing their diabetes.			
**C5**	Diabetes distress occurs when a doctor does not take a patient's concerns seriously and does not provide clear enough directions on how to manage a patient's diabetes			
**C6**	Diabetes distress occurs when a patient with diabetes feels that family or friends do not understand how difficult it is for them to deal with diabetes and are not supportive			
**C7**	Patients experience diabetes distress when they do not understand why their blood sugar levels keep increasing despite eating correctly or adhering to their diabetic medications, and subsequently feel like giving up			

ID Number:			

No.	Questions	True	False	Don't Know
**CONSEQUENCES OF UNTREATED DIABETES DISTRESS**				
**C8**	Diabetes distress can lead to poorer control of diabetes.			
**C9**	Diabetes distress does not affect a person's health-related quality of life.			
**C10**	Diabetes distress does not affect medication adherence.			
**C11**	Diabetes distress may lead to depression.			
**C12**	Diabetes distress may lead to poorer self-care (eg: diet, exercise)			
**DIABETES DISTRESS MANAGEMENT**				
**C13**	Diabetes distress cannot be screened using questionnaires.			
**C14**	A patient should be screened for diabetes distress if their glycaemic control remains persistently poor.			
**C15**	Diabetes distress must be screenedwhen a patient with diabetes has onset of diabetic complications			
**C16**	Addressing and talking about a patient's specific areas of concerns in managing their diabetes can help reduce diabetes distress.			
**C17**	Diabetes distress is highly responsive to interventions that enhance diabetes self-management.			
**C18**	All patients with diabetes distress need to be referred to a psychologist.			

This is the end of the questionnaire. Thank you for your participation.
